# Alleviation of mutant TDP-43-mediated neuropathology by inducible stem cells in monkeys

**DOI:** 10.7150/ijbs.122557

**Published:** 2026-01-01

**Authors:** Xichen Song, Caijuan Li, Yang Yang, Chunhui Huang, Min Chen, Song Lin, Zhonghai Huang, Wei Wang, Kai Liao, Huiyi Wei, Lu Wang, Hao Xu, Yizhi Chen, Yingqi Lin, Jiawei Li, Zhen Dai, Wenguang Xie, Xiao Zheng, Jianhao Wu, Jiale Gao, Jiaxi Wu, Zhuchi Tu, Libing Zhou, Lu Huang, Chaoran Ren, Kwok-Fai So, Peng Yin, Huiming Yang, Shihua Li, Liangxue Lai, Xiao-Jiang Li, Sen Yan

**Affiliations:** 1 State Key Laboratory of Bioactive Molecules and Druggability Assessment, Guangdong Basic Research Center of Excellence for Natural Bioactive Molecules and Discovery of Innovative Drugs, Guangdong Provincial Key Laboratory of Non-human Primate Research, Guangdong-Hong Kong-Macau Institute of CNS Regeneration, School of Medicine, Jinan University, Guangzhou,510632, China.; 2 Guangdong Provincial Key Laboratory of Stem Cell and Regenerative Medicine, Guangzhou Institutes of Biomedicine and Health, Chinese Academy of Sciences, Guangzhou, 510632, China.; 3 South China Institute of Large Animal Models for Biomedicine, Wuyi University, Jiangmen, 529000, China.; 4 Department of Physiology School of Medicine, Jinan University, Guangzhou, 510632, China.; 5 The First Affiliated Hospital of Jinan University, Guangzhou, 510632, China.; 6 Sun Yat-sen University, Guangdong Provincial Key Laboratory of Diagnosis and Treatment of Major Neurological Diseases, Guangzhou, China.; 7 Lingang Laboratory, Shanghai, 201306, China.

**Keywords:** TDP-43 proteinopathies, genetically modified stem cells, NILB-hiPSCs, cynomolgus monkey

## Abstract

Abnormal cytoplasmic accumulation of TAR DNA-binding protein 43 (TDP-43) is a common pathological feature of TDP-43 proteinopathies. Since non-human primate models can better recapitulate this neuropathology than rodents, we used a monkey model to evaluate the therapeutic potential of stem cells for TDP-43-mediated neuropathology. We established a cynomolgus monkey model by expressing mutant TDP-43 (M337V) in the monkey striatum through AAV injection. This model exhibited motor dysfunction and abnormal cytoplasmic TDP-43 accumulation. Using multi-gene modified stem cells (NILB-hiPSCs) that can be induced to differentiate *in vivo* with doxycycline treatment, we found that transplanted NILB-hiPSCs improved the limb movements of the TDP-43-injected monkeys, differentiated into mature neurons, and were integrated with neural circuit activity in the monkey brain. Furthermore, NILB-hiPSC therapy reduced reactive gliosis and diminished the abnormal cytoplasmic localization of mutant TDP-43. These results highlight the potential of *in vivo* inducible stem cells for the treatment of TDP-43 proteinopathies.

## Introduction

The cytoplasmic accumulation of TDP-43, a TAR DNA-binding protein of 43 kDa, in various brain regions is a neuropathological feature in Amyotrophic lateral sclerosis (ALS) and other neurological disorders 1. TDP-43 is encoded by the TARDBP gene and is a DNA/RNA binding protein that normally localizes in the nucleus and plays multiple roles in mRNA splicing, RNA transport, and translation 2-4. Although TDP-43 mutations are rare and only account for 4% of familial ALS cases, almost all ALS cases exhibit pathological features of TDP-43 mislocalization in the cytoplasm and nuclear TDP-43 depletion 5-7. Such cytoplasmic mislocalization is believed to be the cause of loss of normal nuclear function of TDP-43 and acquisition of cytoplasmic toxic function, leading to neuronal death 8, 9. Moreover, cytoplasmic mislocalization of TDP-43 has been observed in diverse brain regions across a spectrum of neurodegenerative diseases 5, suggesting that TDP-43-associated neuropathology is widely implicated in various pathological conditions.

In the majority of mouse models of ALS, transgenic TDP-43 is predominantly found in the nucleus 10-12, while in the TDP-43 transgenic pig model, pathological features of TDP-43 mislocalization in the cytoplasm and nuclear depletion similar to those observed in patients are displayed 13. Additionally, previous studies have shown that in non-human primate models of ALS, local injection of TDP-43 resulted in its predominant localization in the cytoplasm 14, 15. These findings suggest that non-human primate models can serve as a platform for evaluating therapies targeting TDP-43-triggered neuropathology.

Stem cell transplantation has emerged as a potent new strategy to treat a variety of degenerative diseases that currently lack specific or effective treatments 16. This potential stems from the notable plasticity of stem cells and their capacity to differentiate into diverse neuronal types 17. By modulating the host microenvironment to inhibit inflammation, replacing damaged neurons, and exerting required therapeutic effects, stem cell therapy holds great promise 18. However, challenges such as cell transplantation techniques and immunosuppression remain in stem cell research 19, 20. In the context of stem cell therapy for ALS, various cell types are considered, including neural stem cells (NSCs), mesenchymal stem cells (MSCs), glial-restricted progenitor cells (GRPs), embryonic stem cells (ESCs), and induced pluripotent stem cells (iPSCs) 21. In the SOD-1 transgenic rats, both hNSCs and hESCs have demonstrated advantageous outcomes, delaying disease onset and progression 22-25. However, the limited efficiency of neuronal differentiation and ethical issues constrain the extensive application of hNSCs and hESCs 26, 27. MSCs have attracted attention due to their low immunogenicity and their capacity to elevate Glial cell line-derived neurotrophic factor (GDNF) levels following transplantation 28, but their ability to differentiate into neurons remains to be defined 29. Since glial dysfunction critically contributes to neuropathology 30-32, hGRP transplantation has been considered as a potential therapeutic approach to replace pathological glial cells. Nevertheless, a study employing human fetal forebrain hGRP transplantation found no observable therapeutic benefits 33. The low efficacy of stem cell therapy can often be attributed to the host environment's immunogenic reaction towards the transplanted cells.

Human induced pluripotent stem cells (hiPSCs), which have low immunogenicity and ability to survive long-term in cynomolgus monkeys, have also been widely used for stem cell therapy 34, 35. The transplantation of iPSC-derived dopaminergic progenitor cells into a Parkinson's disease (PD) monkey model resulted in their differentiation into mature dopaminergic neurons, substantially improving motor and depressive behaviors in the PD monkey model 36, 37. However, when iPSCs were differentiated *in vitro* and subsequently transplanted into animal models, the survival rate of the transplanted neurons was very low 38.

In the current study, we used multi-gene-modified stem cells, namely NILB-hiPSCs, which have integrated expression cassettes encoding neuronal transcription factors Ngn2, Isl1, Lhx3, and a tetracycline-inducible expression system (Tet-On). These components actively induce *in vivo* differentiation of transplanted stem cells 39*.* Since TDP-43-mediated neuropathology occurs in various brain regions, we assessed the therapeutic effects of NILB-hiPSCs on the cynomolgus monkey striatum and found that NILB-hiPSCs can differentiate and function *in vivo* to alleviate TDP-43-mediated neuropathology, demonstrating for the first time the protective effects of *in vivo* differentiated NILB-hiPSCs on neurodegeneration.

## Materials and Methods

### Ethics statement

The animal use and experiments followed the protocol that was approved by the Institutional Animal Care and Use Committee (IACUC) of Guangdong Huazhen Biotechnology Co., Ltd (Approval No.: HZ2022016-1). This study was conducted in strict compliance with the “Guide for the Care and Use of Laboratory Animals of the Institute of Laboratory Animal Science (est. 2006)” and “The use of non-human primates in research of the Institute of Laboratory Animal Science (est. 2006)” to ensure the personnel safety and animal welfare.

### Animal samples

The experimental cynomolgus monkeys (n=16) used in the study were all housed at Guangdong Huazhen Biotechnology Co., Ltd., which is an Association for Assessment and Accreditation of Laboratory Animal Care (AAALAC)-accredited facility. The cynomolgus monkeys used in this experiment were male individuals aged 4 to 6 years.

### Virus and NILB-hiPSCs

The virus expressing TDP-43 (M337V) is derived from pUAS-hTDP-43 (M337V) plasmid 40, which encodes human mutant TDP-43 (M337V) under the control of the ubiquitin C (UBC) promoter. The vector was packaged by PackGene Biotech with the AAV9 serotype. Purified viruses were stored at -80°C. The genomic titer of the purified viruses (vg) (approximate 10^13^ vg/mL) was determined by PCR method. The control AAV-RFP vector consisted of the same vector as for AAV-TDP-43 and contained the same ubiquitin promoter. The NILB-hiPSCs used in this study were kindly provided by Professor Liangxue Lai from the Guangzhou Institutes of Biomedicine and Health. These cells were generated using previously established methods 41.

### Stereotaxic injection

Using an AAV-9 vector with a human ubiquitin C promoter controlling mutant TDP-43 (M337V) expression, we stereotactically injected the virus into the striatum of cynomolgus monkeys. To investigate different levels of phenotypes, we divided the experiment into five groups: the RT group (n=5) that received AAV-RFP and AAV-TDP-43 injections into the left and right striatum, respectively; the RT-Ctrl group (n=3) that served as the control group and received AAV-RFP injections into the right striatum; the TT group (n=2) that received AAV-TDP-43 injections into both sides of the striatum; the TT-Ctrl group (n=3) that served as the control group and received AAV-RFP injections bilaterally into the striatum, and the wild-type (WT) group (n=3) with no virus injection.

We first administered intraperitoneal injections of atropine (0.3-0.5 mg/kg), ketamine (10-12 mg/kg), and pentobarbital sodium (15-20 mg/kg) to anesthetize the animals, and then immobilized them on the operating table using a David Kopf stereotaxic instrument for virus injection. Prior to injection, we used MRI localization techniques to determine the injection site and needle insertion depth, then injected twenty microliters of virus (total 4 x10^11^v.g.) in two locations in the striatum (including the caudate nucleus and putamen). Two months after injection, behavioral analysis was performed on the injected animals.

### Cell transplantation

The NILB-hiPSCs were stereotactically transplanted to the site of AAV-TDP-43 injection after the onset of motor dysfunction in the cynomolgus monkey model of TDP-43. The needle was inserted into the target brain region at a speed of 4 mm/min and maintained for 5 mins. Subsequently, 20 μl of NILB-hiPSC suspension (~100,000 cells/μl, total 2 million) was injected into the target area of the brain at a rate of 2 μl/min, respectively. The needle was kept in place for 10 mins after transplantation, and then slowly withdrawn at a rate of 4 mm/min. Doxycycline (50 mg/kg) was administered via intraperitoneal injection at intervals of every other day, comprising a total of five injections, to induce differentiation *in vivo*. To reduce immune responses caused by NILB-hiPSCs and control, Cyclosporine A was administered to all monkeys starting three days before cell transplantation, with a dose of 30 mg/kg gradually decreasing to 15 mg/kg until euthanasia.

### Motor Evoked Potential (MEP) experiment

Firstly, the monkey was anesthetized and the fur on its brain and hindlimb gastrocnemius muscle was shaved using an animal hair clipper. Then, the monkey was placed on a surgical table and the receiving electrodes were inserted into the hindlimb gastrocnemius muscle and lateral plantar muscle of the foot, with another ground wire inserted into the subcutaneous tissue of the back. Next, the transcranial magnetic stimulator (Magstim200^2^, Magstim, UK) was set to a stimulation intensity of 60%, and the motor cortex of the brain was stimulated (non-invasively) while recording the electromyographic response of the monkey's hindlimb gastrocnemius muscle. The MEP signals were collected using a multi-channel system (VikingQuest EMG/EP System, Nicolet, USA) and formed into an electromyogram. The hindlimb gastrocnemius muscle was tested at least three times on each side, with a 3-minute interval between each test. Finally, the data from multiple experiments were analyzed and the average value was calculated.

### Grip strength test

To evaluate weakness of the upper limb muscles, we measured grip strength using a spring hand dynamometer connecting with a small handle made specially for measuring monkey upper limb strength (AiDebao HandPink, China). In this test, a monkey was allowed to grasp the handle with one upper limb (left or right), and its muscle strength was measured by the examiner pulling the spring hand dynamometer until the animal released the handle. The digital score of the force on the grip strength meter was recorded for statistical analysis.

### Immunofluorescence staining and analysis

Euthanasia was performed on the monkeys after completion of the behavioral analysis, and their brain tissues were collected for examination. To analyze the differentiation of NILB-hiPSCs in monkey brains, we performed immunocytochemical studies using AAV-TDP-43 or AAV-RFP monkeys. Animals were anesthetized and perfused with 1000 ml of 0.9% NaCl followed by 500 ml of 4% paraformaldehyde in 0.1 M PBS through the left ventricle. Brains were collected and post-fixed in the identical solution for 72 hours, subsequently transferred to a 30% PBS-buffered sucrose solution for 36 hours at 4℃. Following dehydration, brains were sectioned into 30 µm sections using a cryostat (Leica CM1850) at - 20 °C. Brain slices were fixed in 4% paraformaldehyde in PBS for 10 min, permeabilized with 0.2% Triton X-100 in PBS for 30 min, blocked with 3% normal donkey serum in 3% BSA for 1 h, and incubated with primary antibodies (anti-GFP abcam, #ab13970; anti-Flag Sigma, #F1804; anti-STEM121 TAKARA, #Y40410; anti-DCX abcam, #ab18723; anti-NeuN abcam, #ab104224; anti-GFAP Invitrogen, #13-0300; anti-Iba1,Wako, #019-19741; anti-Tuj1, Cell Signaling Technology, #568S; anti-MAP2 abcam, #ab96378; anti-SOX2 R&D, #MAB2018; anti-Ki67 abcam, #ab16667) overnight at 4℃ in 3% BSA. After several washes with PBS, brain sections were incubated with Alexa 555/488-conjugated secondary antibody. Nuclei were labeled with 0.01 μg/ml DAPI. Whole brain images were captured with an automatized slide scanner (TissueFAXS Whole Slide Scanner; TissueGnostics, Vienna, Austria) fitted with 20X objectives. Confocal images were collected with FV3000 (OLYMPUS) confocal laser scanning microscope.

Since the transplanted NILB-hiPSCs were labeled with GFP, we identified brain regions in the monkeys that showed GFP signals. These regions were then sectioned and immunostained. The brain region was cut into 30 μm sections. For each section, four to five images were captured. The number of cells that were labeled by specific antibodies per image was counted. The data were derived from sections from at least two animals and are presented as mean ± SEM for statistical analysis.

### H&E staining and quantification

The gastrocnemius muscle of monkeys was immersion-fixed in muscle fixative solution for 48 h, cryoprotected in 30% sucrose and sectioned at 5 μm thickness. HE staining was performed by Servicebio (Wuhan, China). Microscopic images were acquired using TissueFAXS PLUS tissue analysis system (TissueGnostics). The fiber size distribution is typically quantified in terms of the minimum Feret diameter, as it is the least affected by distortion due to oblique cross-sectioning of muscle tissue 42. We randomly collected a minimum of 1,000 individual muscle fibers from the gastrocnemius muscles of monkeys in the RT-Ctrl (RFP), RT and TT (TDP-43), and RT treatment (NILB-hiPSCs) groups to quantify their minimum Feret diameters. To investigate the histological changes in muscle sections, the minimum Feret diameters of muscle fibers were quantitatively assessed using ImageJ (https://imagej.net/). The data were then imported into GraphPad Prism 8 for statistical analysis to evaluate muscle atrophy.

### Electrophysiological recordings

To prepare brain slices, animals were deeply anesthetized with intravenous sodium pentobarbital (30 mg/kg). The brain tissue was rapidly dissected and coronal sections (250 μm thick) containing the graft were obtained using a vibratome (VT1200S; Leica Microsystems) in ice-cold artificial cerebrospinal fluid (ACSF: 119 mM NaCl, 2.5 mM KCl, 1 mM sodium phosphate buffer, 11 mM glucose, 26.2 mM NaHCO_3_, 2.5 mM CaCl_2_, and 1.3 mM MgCl_2_, pH 7.4, 290 mOsm). The brain slices were incubated at 35±1°C for 1 hour and then allowed to recover for 1 hour at room temperature in ACSF. Subsequently, a single slice was placed into the recording chamber and continuously perfused with oxygenated ACSF at a rate of ~2 ml/min. The pipettes (P-97, Sutter Instruments), with resistance ranging from 3 to 6 megohms, were filled with intracellular solution containing the following: for voltage clamp: 130 mM CsMeSO4, 10 mM NaCl, 10 mM EGTA, 4 mM MgATP, 0.3 mM Na_3_GTP, and 10 mM Hepes, 290 mOsm, adjusted to 7.4 with CsOH; for current clamp: 135 mM KMeSO4, 10 mM KCl, 10 mM Hepes, 10 mM Na2-phosphocreatine, 4 mM MgATP, and 0.3 mM Na_3_GTP, 290 mOsm, adjusted to 7.4 with KOH.

The neurons induced by NILB-hiPSCs were subjected to whole-cell patch-clamp recordings under IR-DIC visualization (Zeiss, Axioskop 2). The recordings were obtained using a MultiClamp 700B amplifier and a 1440A digitizer (Molecular Device). To evaluate the neuronal excitability in current clamp mode, a series of depolarizing pulses (ranging from 0 to 100 pA, in 10 pA increments) were applied. In voltage clamp mode, spontaneous excitatory postsynaptic currents (sEPSCs) were recorded while the membrane potentials were clamped at -70 mV. The traces were low-pass filtered at 2 kHz and digitized at 10 kHz. After obtaining stable whole-cell recordings with an access resistance below 25 megohms, basic electrophysiological properties were recorded. Clampfit 10.0 software was used for offline data analysis.

### MRI data acquisition

MR anatomical images were acquired on a 3.0 T scanner (GE Discovery 750, Milwaukee, USA) equipped with an 8-channel customized head coil for macaques (Medcoil MK80, Suzhou, China) at the PET/CT-MRI center in the First Affiliated Hospital of Jinan University.

Prior to each MRI scan, the monkeys underwent a fasting period of at least 6 hours and were anesthetized with an intramuscular injection of ketamine (0.1-0.2 mL/kg) and atropine (0.1 mL/kg). The whole-brain images were acquired with a 3D Bravo T1 sequence (TR=8.4ms, TE=3.5ms, slice thickness=0.5mm, matrix size=300 × 300, FOV=15×15 cm).

### PET data acquisition and data analysis

Animals underwent PET with [18F]LW223 to assess in* vivo* neuroinflammation levels in the striatum. [18F]LW223 was produced at Center of Cyclotron and PET Radiopharmaceuticals, Department of Nuclear Medicine and PET/CT-MRI Center, The First Affiliated Hospital of Jinan University, according to the procedures from reported literature 43. Each monkey was first anesthetized with ketamine (0.1-0.2 mL/kg) and atropine (0.1 mL/kg) and then intravenously injected with a dose of 0.3-0.6 mCi/kg of [18F]LW223. After 60 minutes, the monkey was placed in a homemade PET imaging head holder and underwent a 10-minute static brain PET/CT scan while in the supine position.

All data were acquired using a PET/CT system (GE Discovery Elite 690, USA). CT and PET data were collected with a slice thickness of 3.27 mm, slice interval of 3.75 mm, matrix size of 256 × 256 and scan FOV of 70 cm in 3D time-of-flight (TOF) mode. CT data, acquired for attenuation-corrections, were reconstructed in standard mode with DFOV of 30 cm and window width/window level 100/45, advanced statistical iterative reconstruction 40%. The PET data were attenuation-corrected by integrated CTAC technology. PET-MR images were co-registered by PMOD 4.1 (PMOD technology, Switzerland) for analyzing the imaging data in the volumes of interest (VOIs), including the caudate, the putamen and the cerebellum cortical grey matter. Standard uptake values (SUV) were calculated as SUV = [(VOI activity) × (bodyweight)]/ injected dose), and the SUV ratio (SUVr) was calculated using the cerebellum cortical grey matter as a pseudo reference region 44.

### Quantification and statistical analysis

Statistical significance was assessed using the two-tailed Student's t test for comparing two groups. When analyzing multiple groups, we used one-way ANOVA to determine statistical significance. For monkey that was repeatedly subjected to behavioral tests, we analyzed the data using two-way ANOVA, followed by Bonferroni post-hoc comparison. Data are mean ± SEM. GraphPad Prism ver 8.0.2 package (GraphPad Inc, USA) was employed for statical analysis and data plotting.

## Results

### Overexpression of TDP-43 (M337V) resulted in impaired muscle function and pathological features in cynomolgus monkeys

In our study, we assessed the therapeutic effects of stem cells on TDP-43-mediated neuropathology in the striatum. Since TDP-43-mediated neuropathology is not limited to the spinal cord, we chose to characterize the striatal neurons for several reasons. Firstly, TDP-43 has been shown to mediate neuropathology in various brain regions 45, 46. Therefore, studying striatal neuropathology can serve as a representative model for TDP-43-mediated neurotoxicity. Additionally, the striatum is easily identifiable through MRI, making it more convenient to deliver AAV (adeno-associated virus) into this specific brain region compared to regions containing motor neurons. Secondly, assessing neurodegeneration in the striatum can be more readily achieved using MRI than in the spinal cord. Thirdly, striatal degeneration can result in noticeable limb movement difficulties, which provides a clear basis for evaluating the therapeutic effects on animal behaviors 12, 14. Lastly, after stem cell therapy, the electrophysiological function of striatal neurons in cultured brain sections can also be examined.

**Figure 1:**
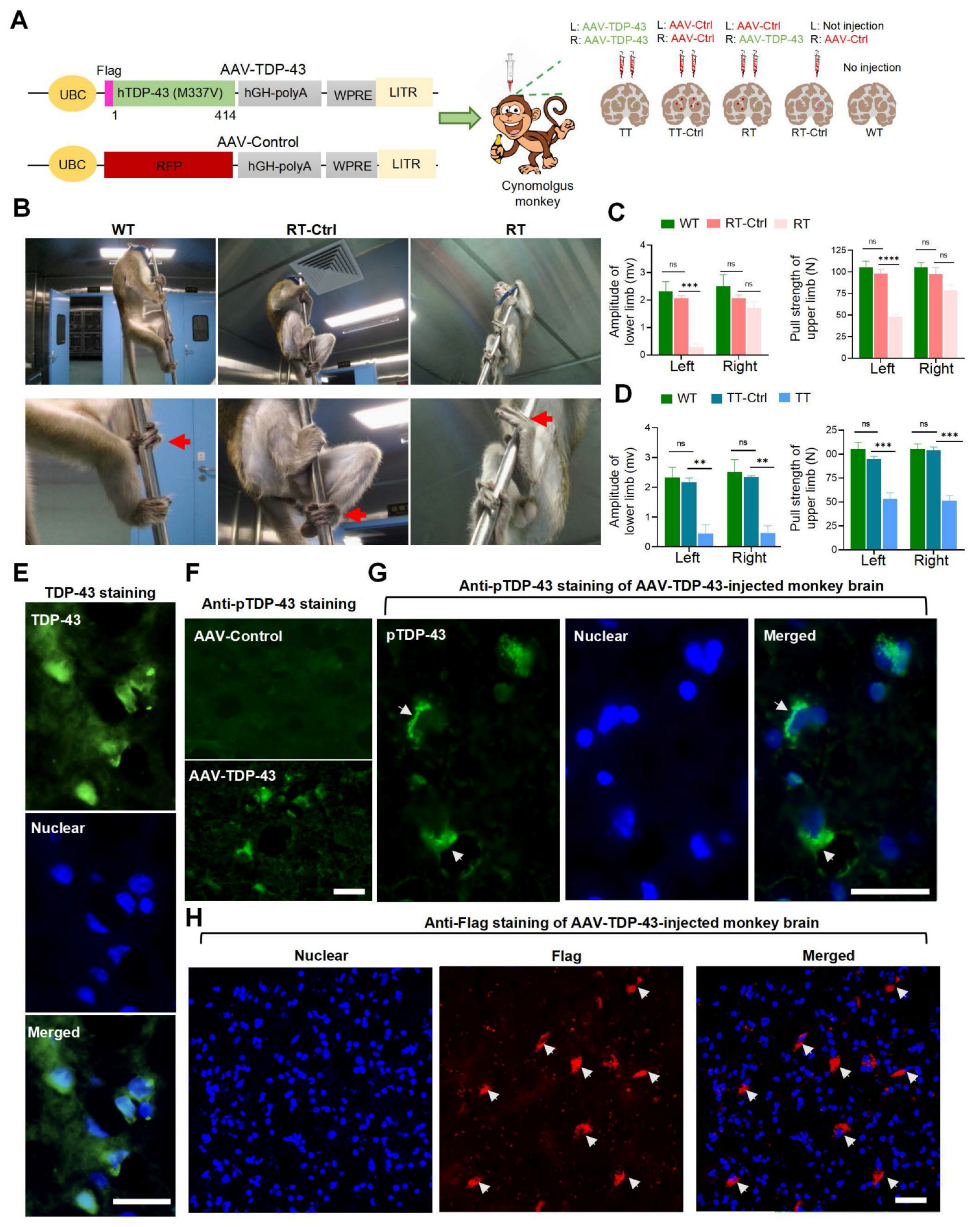
Behavioral and pathological evaluations of AAV9-TDP-43-injected monkeys. (A) The schematic diagram illustrates the injection protocol of the AAV9-Control RFP (AAV-Ctrl) or AAV9-TDP-43 virus that expresses mutant TDP-43 (M337V). (B) Representative photographs depict a WT group monkey, an RT-Ctrl group monkey, and an RT group monkey (from left to right). The monkey in the RT group displays paralysis of the left lower extremity, as indicated by an arrow, which is controlled by the contralateral right side of the striatum. (C) The amplitude of the MEP in the gastrocnemius muscle of the lower limb (left panel), as well as the grip strength of the upper limb (right panel), exhibited a significant reduction in RT group monkeys compared to those in the RT-Ctrl group and the WT group (n=5 monkeys from RT group, n=3 monkeys from RT-Ctrl group, n=3 monkeys from WT group). (D) The amplitude of the MEP in the gastrocnemius muscle of the lower limb (left panel), as well as the grip strength of the upper limb (right panel), exhibited a significant reduction in TT group monkeys compared to those in the TT-Ctrl group and the WT group (n=2 monkeys from TT group, n=3 monkeys from TT-Ctrl group, n=3 monkeys from WT group). (E) TDP-43 immunostaining showing the selective nuclear and cytoplasmic distribution of TDP-43 in the AAV-TDP-43-injected brain region. (F) The antibody to the pathological phosphorylated TDP-43 (pTDP-43 labeled transgenic TDP-43 in the AAV-TDP-43-injected brain region. (G) pTDP-43 immunostaining revealing the cytoplasmic distribution of transgenic mutant TDP-43. The nuclei of cells were labeled with DAPI (blue). (H) Flag immunostaining showed the presence of mutant TDP-43 (indicated by an arrow) in the cytoplasm of monkey striatum four weeks after injection. Arrows indicated cytoplasmic TDP-43. Scale bars, 40 μm. ns, no significant difference; ∗∗p < 0.01; ∗∗∗p < 0.001; ∗∗∗∗p < 0.0001. All data are presented as mean ± SEM.

We generated a TDP-43 primate model by administering the TDP-43 (M337V) virus via unilateral or bilateral injections into the striatum of cynomolgus monkeys. To serve as a control, we used the AAV-RFP virus for unilateral or bilateral injection. (Fig 1A). Two months after viral injection, monkeys in the RT group (n=5) exhibited a diminished ability of their left limbs to grasp the bars, indicative of impaired function of their right striatum caused by the mutant TDP-43, as compared to the RT-Ctrl (n=3) group and the WT group (n=3) (Figure 1B). In the Motor Evoked Potential (MEP) experiment, it was observed that TDP-43-injected monkeys from the RT group showed varying degrees of decreased amplitudes of the gastrocnemius muscle action potential in the left hind limb (Figure 1C, left panel). The grip strength test revealed that the RT group demonstrated a significant decline in the pull strength of the left upper limb, which is controlled by the right striatum, compared to the RT-Ctrl and WT monkeys (Figure 1C, right panel). The TT group (n=2) monkeys who received bilateral injections of AAV-TDP-43 demonstrated significant reductions in the amplitudes of MEP in the gastrocnemius muscles of both legs (Figure 1D, left panel). Additionally, in the grip strength tests, the bilateral injection monkeys showed a marked decrease in pull strength in both arms compared to the TT-Ctrl group (n=3) and WT group monkeys (Figure 1D, right panel). These findings provide compelling evidence that overexpression of TDP-43 (M337V) in the striatum resulted in neuronal damage and toxicity.

In almost all cases of ALS, a major pathological feature is the mislocalization of TDP-43 from the nucleus to the cytoplasm 1. To investigate the subcellular localization of TDP-43 (M337V) in the brains of monkeys, we conducted immunofluorescence staining using an antibody against a C-terminal peptide corresponding to residues surrounding Gly400 of human TDP-43. This antibody could detect the nuclear expression of normal TDP-43 in the monkey brain (Figure S1) but is difficult to distinguish between the nuclear TDP-43 and cytoplasmic distribution of transgenic TDP-43, which is expressed under the control of the UBC promoter in AAV9 (Figure 1E). We then performed immunostaining with the antibody against phosphorylated TDP-43 (S409/410), which specifically labels the pathological form of TDP-43 in the AAV9-TDP-43 injected monkey brain (Figure 1F) and clearly detected the cytoplasmic mutant TDP-43 (Figure 1G). To validate this finding, we used the anti-Flag antibody, which specifically labels transgenic mutant TDP-43, and found that mutant TDP-43 formed cytoplasmic aggregates in the AAV-TDP-43-injected monkey striatum (Figure 1H). By employing multiple antibodies, we thus confirmed the cytoplasmic distribution of mutant TDP-43 in the monkey brain, which is consistent with observations made in the brains of patients 46 and transgenic monkeys expressing mutant TDP-43 14. Our monkey model, therefore, recapitulates significant pathological features of cytoplasmic TDP-43, along with associated phenotypes related to movement, enabling us to assess the therapeutic potential of NILB-hiPSCs transplantation.

### NILB-hiPSCs ameliorated movement dysfunction in TDP-43 (M337V) monkey model

**Figure 2:**
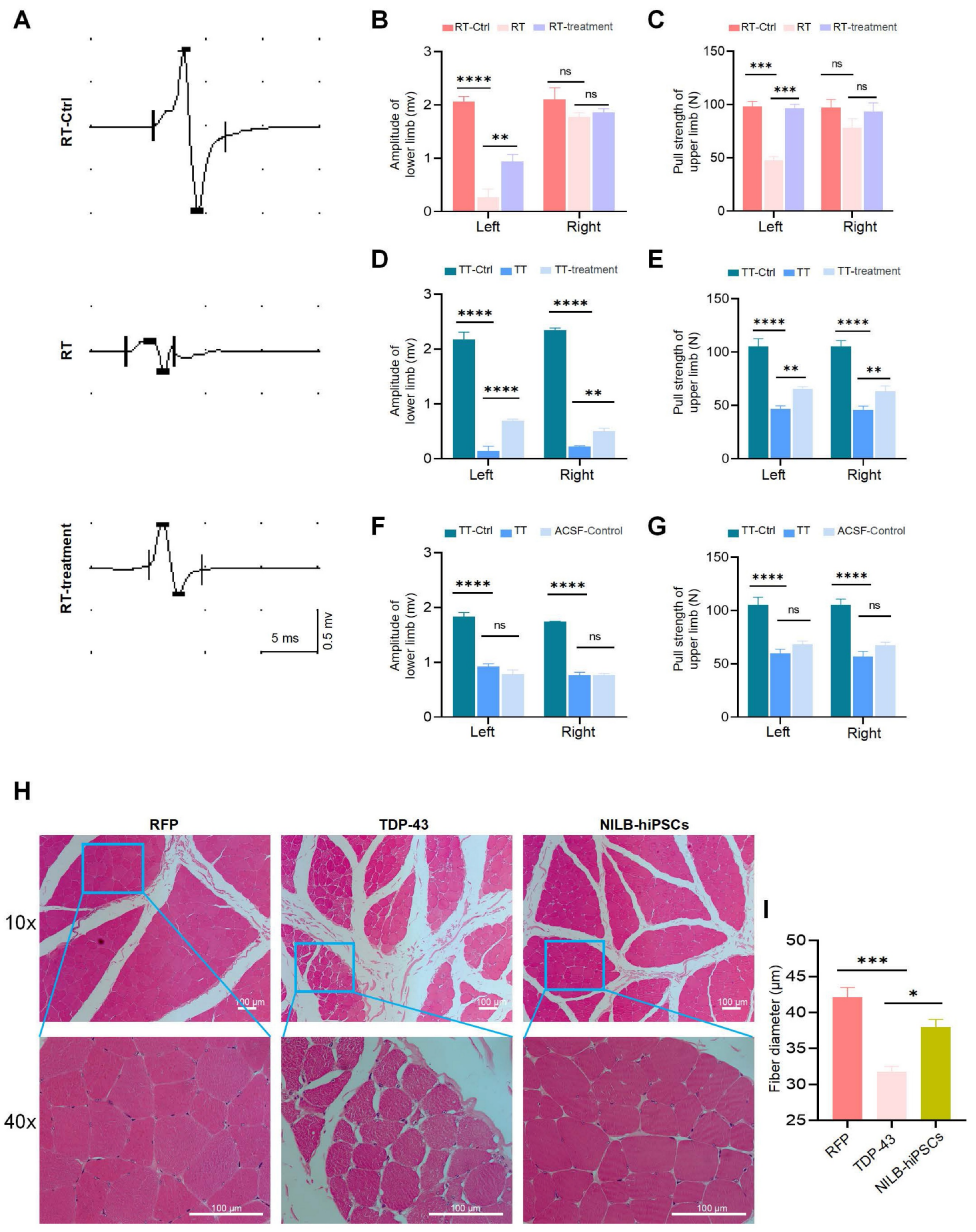
Effects of NILB-hiPSCs treatment on limb function in TDP-43 Monkeys. (A) The amplitude of MEP was measured in the gastrocnemius muscle of a RFP monkey, a TDP-43 monkey, and a TDP-43 monkey with NILB-hiPSCs transplantation. (B) Quantitative analysis showed that the amplitude of the left lower limb was significantly increased after NILB-hiPSCs transplantation into the right striatum of TDP-43 monkeys from the RT group, compared with before treatment and RT-Ctrl group (n=4 monkeys from RT, n=3 monkeys from RT-Ctrl). (C) The pull strength of the left upper limb was also significantly improved after transplantation of NILB-hiPSCs into the right striatum of TDP-43 monkeys from the RT group, as measured by pull strength measurements (n=4 monkeys from RT, n=3 monkeys from RT-Ctrl). (D) Quantitative analysis showed that the amplitude of the lower limb was significantly increased after NILB-hiPSCs transplantation into bilateral striatum in one monkey from the TT group, compared with before treatment. (E) Similarly, the pull strength of the upper limb was significantly improved after transplantation of NILB-hiPSCs into the bilateral striatum of the monkey from the TT group, as measured by pull strength measurements. (F) Amplitude did not change after ACSF transplantation into bilateral striatum in one monkey from the TT group, serving as a control for panel D. (G) Similarly, the pull strength of the upper limb did not change after ACSF transplantation into bilateral striatum in one monkey from the TT group, serving as a control for panel E. (H) H&E staining revealed skeletal muscle atrophy in the TDP-43 monkey compared to the RFP monkey, while NILB-hiPSCs treatment prevented further muscle degeneration. Scale bar, 100 μm. (I) From each group (4 monkeys in the RFP group, 3 in the TDP-43 group, and 4 in the NILB-hiPSCs group), at least 1,000 muscle fibers were randomly collected to quantify the minimum Feret diameters. *P <0.05; ∗∗p < 0.01; ∗∗∗∗p < 0.0001. All data are presented as mean ± SEM.

We developed NILB-hiPSCs, which are multi-gene-modified stem cells expressing several neuronal transcription factors for their differentiation 39, and utilized these cells for treatment. Two months after the injection of AAV9-TDP-43 into the monkey striatum, we transplanted two million NILB-hiPSCs into the striatum of symptomatic TDP-43 cynomolgus monkeys for therapeutic purposes. Subsequently, following three months of treatment involving transplanted NILB-hiPSCs and cyclosporine A administration, the motor function of the TT group monkey showed improvement (Supplementary video). In our prior experiments, transplanting hiPSCs that failed to differentiate under an identical cyclosporine A protocol did not improve motor function in AAV-TDP-43-injected monkeys. Therefore, the motor enhancement observed here is likely a direct therapeutic effect of NILB-hiPSCs rather than a non-specific effect of immunosuppression. Since the gastrocnemius muscle action potential can reflect muscle function and limb's ability to move and grasp 47, 48, we used this measure to evaluate the therapeutic effects of NILB-hiPSCs (Figure 2A). Regarding the assessment of limb function, we observed a significant decrease in the amplitude of MEP in the gastrocnemius muscle of the left hind limb (Figure 2B), as well as in the pull strength of the left upper limb (Figure 2C), in the RT group before treatment, as compared to the RT-Ctrl group. However, these deficits were significantly improved after NILB-hiPSCs therapy (Figure 2B, C).

The positive outcomes observed with the NILB-hiPSCs transplantation were further confirmed in the TT group, where the muscle function was also significantly improved, as illustrated in Figure 2D-G. Moreover, similar to the RT group, the strength of the left and right upper limbs in the TT group was markedly reduced before treatment, as compared to the TT-Ctrl group, but was partially improved after NILB-hiPSCs treatment (Figure 2D, E). Monkeys treated with artificial cerebrospinal fluid (ACSF) exhibited no improvement in muscle function (Figure 2F, G). The H&E staining results showed that the muscle fibers of the TDP-43 monkey exhibited greater atrophy than those of the RFP monkey, but this atrophy was alleviated by NILB-hiPSCs treatment (Figure 2H, I). These findings provide compelling evidence that transplantation of NILB-hiPSCs can effectively enhance muscle function in the TDP-43 (M337V) monkey model.

### NILB-hiPSCs were induced to differentiate into mature neurons

**Figure 3:**
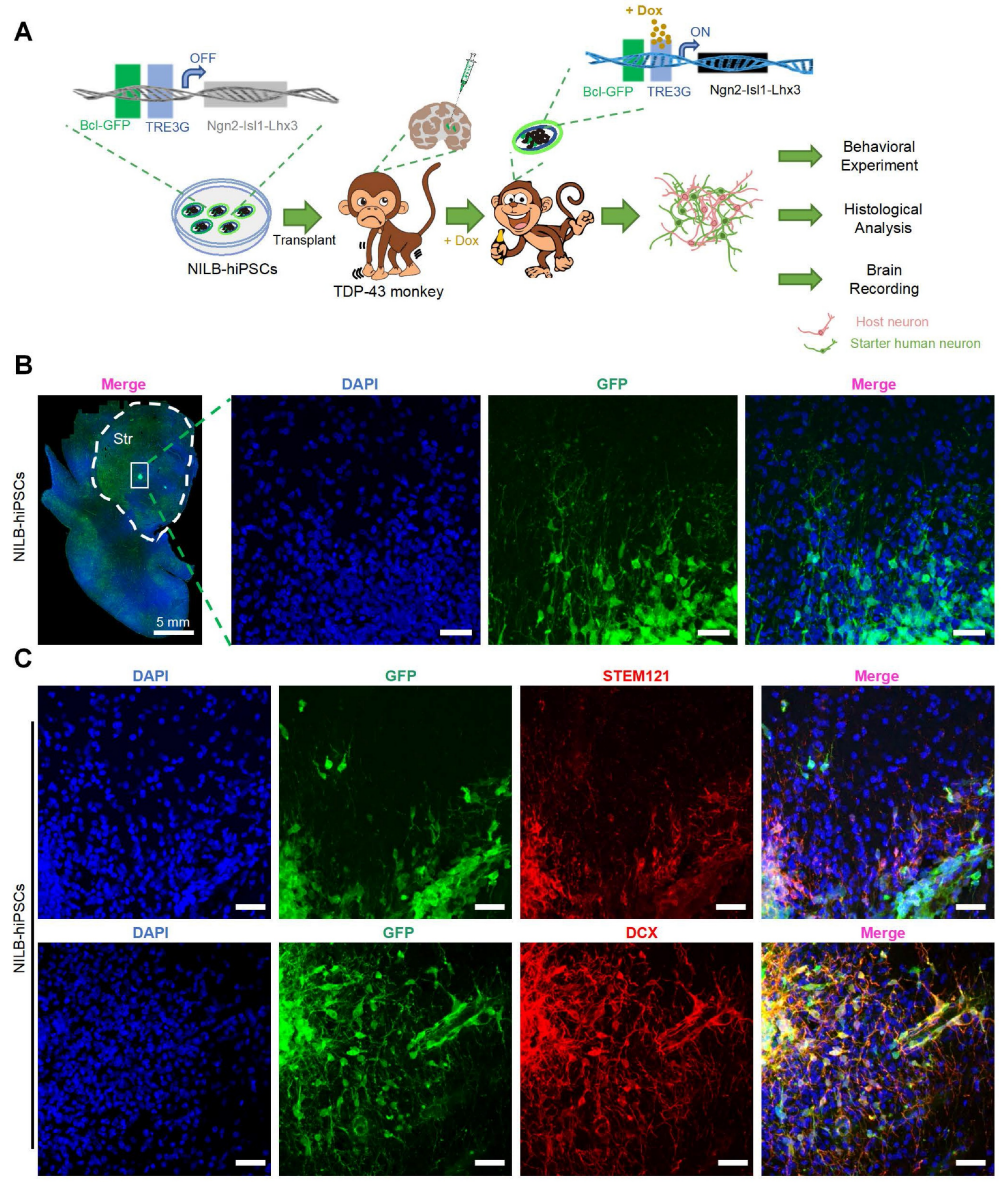
NILB-hiPSCs were differentiated into neurons by Dox in AAV TDP-43-injected monkeys. (A) A strategy for evaluating the therapeutic impact of NILB-hiPSCs transplantation in TDP-43-injected monkeys. (B) Coronal sections of the striatum from the RT group monkey, three months post-transplantation of NILB-hiPSCs, are shown. High magnification reveals GFP immunostaining (green), illustrating the survival and differentiation of NILB-hiPSCs. (C) The TDP-43-injected monkey striatum with NILB-hiPSCs transplantation. Upper panels demonstrate double immunofluorescent staining with antibodies to STEM121 and GFP, whereas lower panels demonstrate co-labeling of DCX and GFP, indicating the differentiation of NILB-hiPSCs into neurons rather than glial cells. Scale bar, 40 μm.

We then evaluated the survival and differentiation of transplanted NILB-hiPSC in cynomolgus monkeys injected with AAV-mutant TDP-43, as illustrated in Figure 3A. As the transplanted cells are confined to discrete regions, stereological analysis is challenging due to its requirement for a large volume of tissue sections. we identified the transplanted NILB-hiPSCs by leveraging their GFP expression and performing immunofluorescence staining. Immunofluorescence analysis of GFP expression demonstrated that the transplanted NILB-hiPSCs exhibited extensive survival in the striatum and displayed a tendency to radiate outward, with long axons projecting in that direction (as illustrated in Figure 3B). We estimated that 70% of transplanted cells near the transplantation site survived three months after transplantation. Furthermore, immunofluorescence analysis using the human cytoplasmic specific antibody STEM121, which has been widely used for identification of human cells 49-51, showed co-localization of GFP-positive cells with STEM121, confirming that the GFP-positive cells were indeed exogenous human cells derived from transplantation process (Figure 3C top). To validate the differentiation of transplanted cells into neurons, we performed doublecortin (DCX) staining, which is a marker for early neuronal development (Figure S2). The results revealed co-localization of GFP-positive cells and DCX, demonstrating that the stem cells had been converted into neurons (Figure 3C bottom). These findings establish that *in vivo* induction using Dox can induce differentiation of NILB-hiPSCs transplanted into neurons in the brains of TDP-43-injected monkeys.

**Figure 4:**
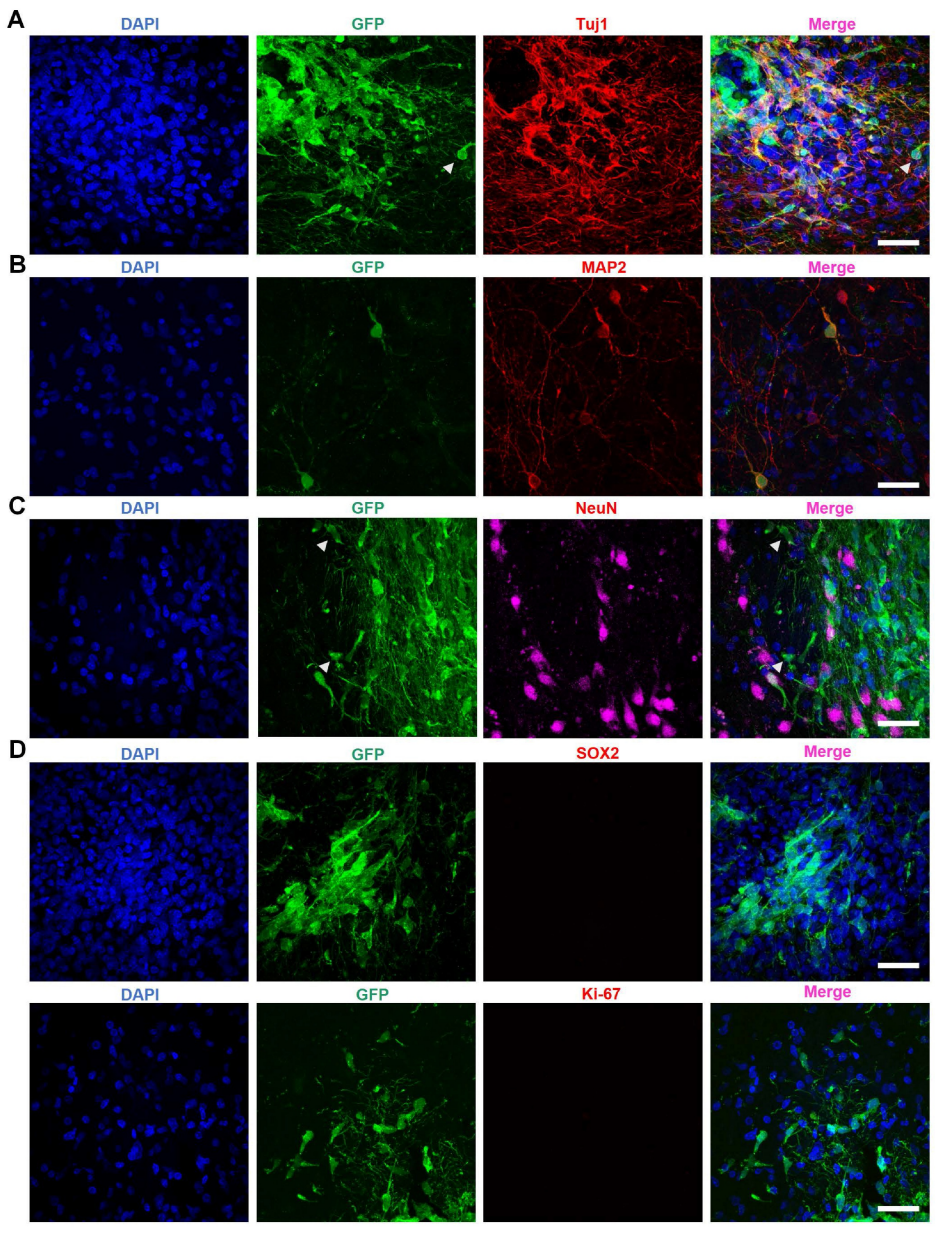
The maturation of NILB-hiPSCs-derived neurons in the stratum of monkeys. (A-C) Immunofluorescence analysis of Tuj1 (A), MAP2 (B), and NeuN (C) expression within the GFP+ grafts. Arrowheads indicate that some GFP-positive cells are negative for Tuj1 and NeuN. (D) Double immunofluorescent staining of transplanted NILB-hiPSCs-derived neurons with antibodies to SOX2 (top panel) or Ki67 (bottom panel) and GFP. Scale bar, 40 μm.

Further investigations revealed the co-localization of the neuronal marker Tuj1 with GFP-positive transplanted cells (Figure 4A). Additionally, transplanted GFP-positive cells expressed MAP2 (Figure 4B) and NeuN (Figure 4C) - both markers for mature neurons - suggesting that NILB-hiPSCs had differentiated into mature neurons. However, we were unable to observe the expression of DARPP-32 in these neurons, suggesting that they do not fully differentiate into mature medium spiny neurons (MSNs) because of the difficulty in using human stem cells to produce D1- and D2-MSNs in the brain 73. Thus, it is important to determine whether the transplanted neurons are functional in the striatum and whether their transplantation does not result in tumor formation.

The prospect of tumor formation in stem cell therapy has always been a critical concern 52, 53. SOX2, a marker protein for cell pluripotency, suggests the possibility of further differentiation and therefore indicates the likelihood of teratoma formation 54. Meanwhile, Ki67 is an active nuclear protein that is present during cell proliferation but disappears once cell growth stops such that It is typically utilized to detect tumor formation 55. To confirm the safety of NILB-hiPSCs transplantation and rule out the possibility of tumor formation, we conducted staining for SOX2 (Figure 4D top) and Ki67 (Figure 4D bottom and Figure S3). The results showed minimal SOX2 and Ki67 positive cells (Figure 4D), suggesting that the transplantation of *in vivo*-induced NILB-hiPSCs in the TDP-43-injected monkeys has low tumor-forming potential and high safety.

### NILB-hiPSCs differentiated into functional neurons

**Figure 5:**
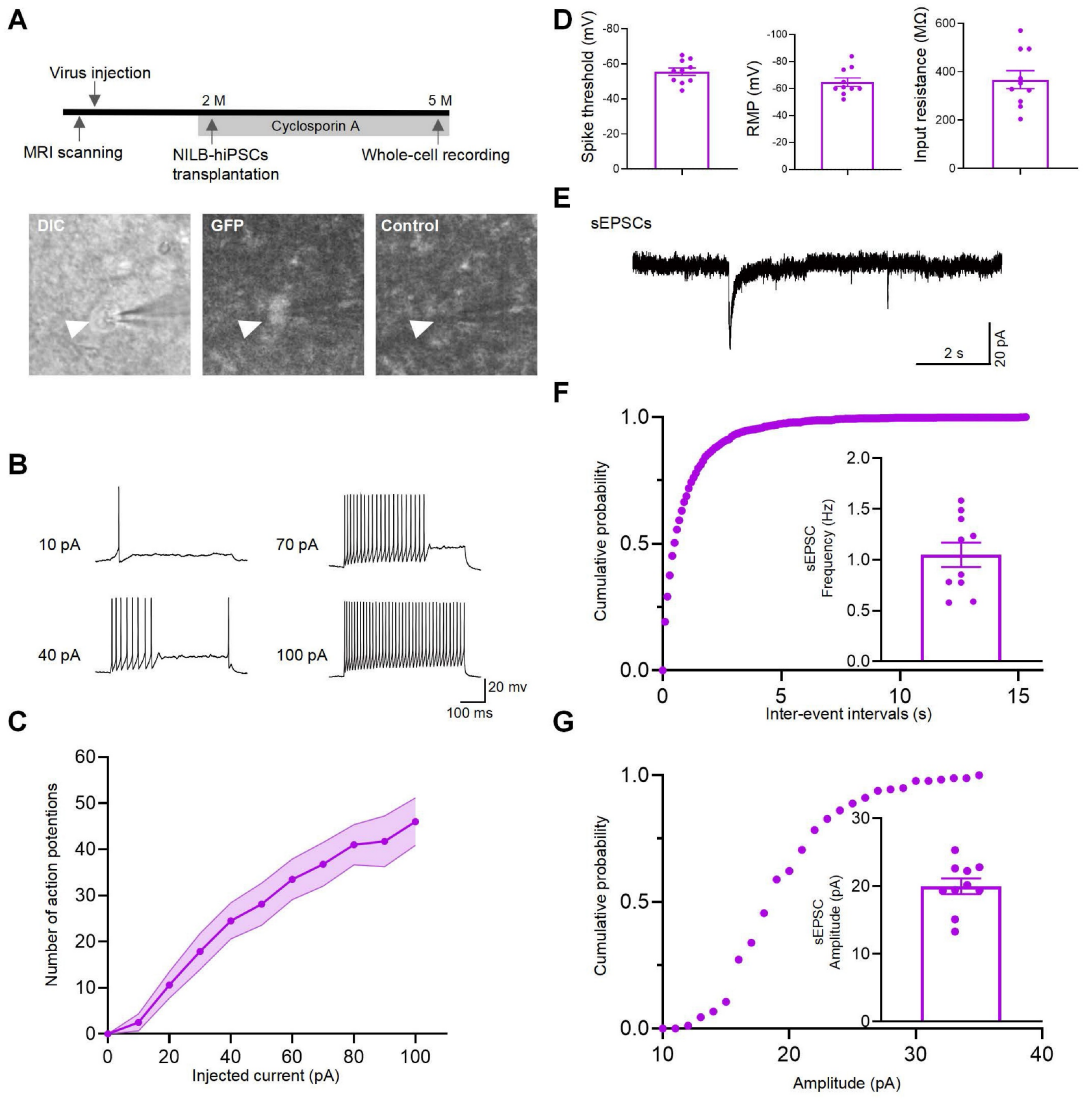
NILB-hiPSCs differentiated into functional neurons in the monkey striatum post-Dox induction. (A) The upper panel presents the experimental diagram of NILB-hiPSC transplantation. The lower panel demonstrates the identification of GFP-positive grafted cells using DIC and fluorescence imaging, with a corresponding background image (without fluorescence excitation) serving as a control. (B) Repetitive firing induced by 10, 40, 70, and 100 pA inward current injections in neurons from GFP-positive grafts of 2 monkeys. (C) The current-evoked action potentials in the transplanted neurons showed a dependence on the current intensity with regards to the frequency of action potentials. (D) Group data of spike threshold, resting membrane potential, and input resistance. (E) Representative traces of sEPSCs from GFP-positive neurons in the TDP-43-injected monkey striatum with NILB-hiPSC transplantation at three months post-transplantation. (F) Cumulative probability plots of sEPSC interevent intervals and average sEPSC frequency (Hz). (G) Cumulative probability plots of sEPSC amplitude and average sEPSC amplitude (pA). The results indicate that the differentiated cells exhibited functional characteristics of mature neurons, including spontaneous postsynaptic currents and action potentials.

To evaluate the functionality of NILB-hiPSC-derived neurons in the monkey brain, electrophysiological measurements were conducted on two monkeys 3 months post-transplantation. To prevent immune rejection, administration of Cyclosporine A is initiated three days before transplantation of NILB-hiPSCs and continues until euthanasia (Figure 5A top). Whole cell patch-clamp recording was performed on GFP-positive grafted cells in brain slices under a fluorescence microscope (Figure 5A bottom). We injected a series of step currents to examine the passive and active membrane properties. The grafted GFP-positive neurons had average input resistance of 367.5 MΩ (n=10 cells), spike threshold of -55.6 mV (n=10 cells) and resting membrane potential of -64.8 mV (n=10 cells) (Figure 5D). These recorded cells showed repetitive firing with increasing step currents, a characteristic feature of mature neurons (Figure 5B). Accordingly, the number of action potential of the GFP-positive neurons showed a gradual increase with increasing current injections from -20 to 100 pA (Figure 5C). In voltage-clamp mode at a holding potential of -70 mV, we observed barrages of synaptic currents (sEPSCs), with median frequency of 1.03 Hz, indicative of the formation of functional synapses onto these recorded cells (Figure 5E-G). In conclusion, patch-clamp techniques were used to record the electrophysiological activity of mature functional neurons derived from NILB-hiPSCs, suggesting that they can differentiate into functional neurons and potentially participate in the neural circuitry of the monkey brain *in vivo*.

### NILB-hiPSCs treatment reversed the cytoplasmic localization of mutant TDP-43

**Figure 6:**
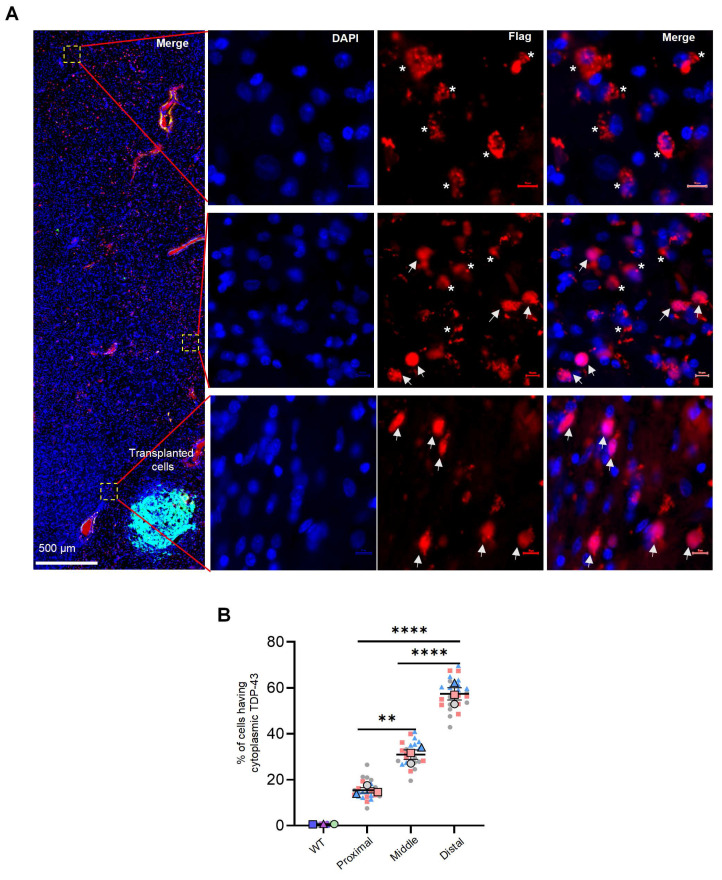
NILB-hiPSCs reversed abnormally cytoplasmic localization of mutant TDP-43. (A) As the distance from GFP-positive transplanted cells increases, mutant TDP-43 exhibits cytoplasmic localization. Arrows indicate the nuclear localization of mutant TDP-43, whereas asterisks indicate its cytoplasmic localization. (B) Superplot analysis of the number of cells displaying cytoplasmic TDP-43 in the proximal, middle, and distal regions surrounding the NILB-hiPSCs transplantation sites. The cell count in each image (40x magnification) was recorded and color-coded to indicate the respective animals (3 monkeys in each group) it originated from. The total cell count (2500-3200) in each animal was determined by counting Dapi-positive nuclei. One-way ANOVA revealed statistical significance (**P < 0.01, ****P < 0.0001). Data are presented as mean ± SEM (n=3).

Cytoplasmic mislocalization of TDP-43, along with the subsequent loss of nuclear TDP-43, are crucial pathological features of ALS and other neuropathological conditions. NILB-hiPSCs therapy reversed the mislocalization of mutant TDP-43. The reversal effects are dependent on the transplanted cells, as more TDP-43 expressing cells near the transplanted cells show the nuclear distribution of TDP-43 whereas those cells that are away from the transplanted cells display the cytoplasmic distribution of TDP-43 (Figure 6A, B). This suggests that NILB-hiPSCs may have the ability to reverse the abnormal cytoplasmic localization of mutant TDP-43 in surrounding neurons by restoring normal cell-cell interactions. This reversal of cytoplasmic TDP-43 localization may be beneficial in mitigating the toxicity of cytoplasmic TDP-43 in the surrounding neurons.

### Treatment with NILB-hiPSCs reduces excessive activation of glial cells induced by TDP-43 (M337V)

**Figure 7:**
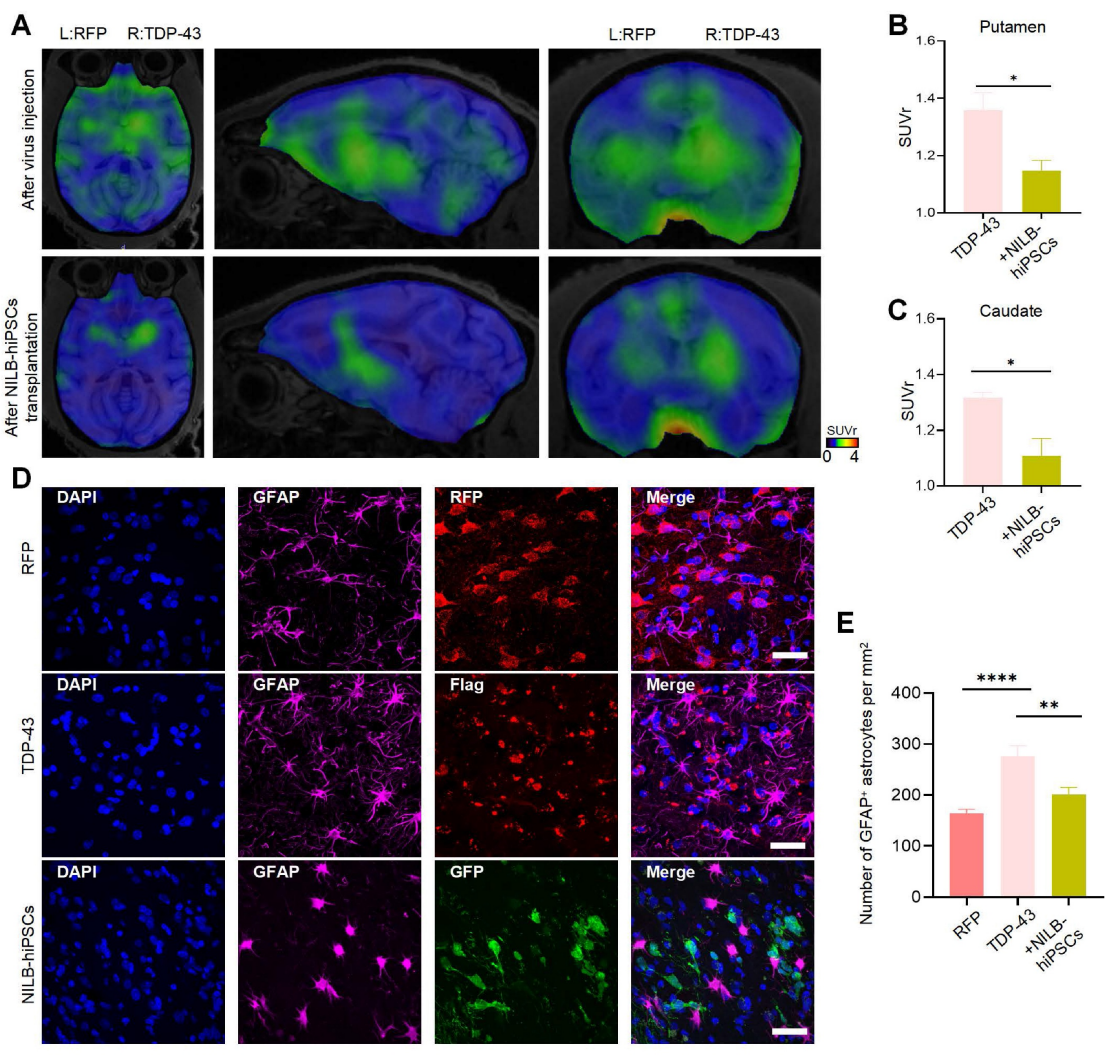
Reducing neuroinflammation in the TDP-43-injected monkey striatum by NILB-hiPSCs. (A) PET/MRI imaging with [18F]LW223 from TDP-43 monkey at pre- and post-transplantation stage of NILB-hiPSCs in the unilateral TDP-43 injected group. (B, C) The quantification of [18F]LW223 binding potential in putamen (B) and caudate (C) from TDP-43 monkey at pre- and post-transplantation stage of NILB-hiPSCs. Data are presented as mean ± SEM. (n = 4 animals). Significance was assessed by a two-tailed unpaired Student's t-test (*P <0.05). (D) Double immunostaining of the striatum of monkey showing that NILB-hiPSCs reduced GFAP-positive cells in TDP-43 monkey with NILB-hiPSCs transplantation. Scale bar, 40 μm. (E) Quantitative assessment of GFAP-positive cells. One-way ANOVA revealed statistical significance (*P < 0.05, **P < 0.01, ****P < 0.0001). Data are presented as mean ± SEM (n=3).

Overactivation of brain glial cells induced by mutant TDP-43 proteins is a significant pathological hallmark of ALS and TDP-43 associated neuropathology 56, 57. Translocator protein (TSPO), an 18 kDa protein that is widely distributed throughout the body 58, is significantly increased in activated microglia and reactive astrocytes. Thus, it serves as a valuable biomarker for neuroinflammation 59. The use of appropriate TSPO positron-emission tomography (PET) ligands enables non-invasive quantification of TSPO, allowing for evaluation of neuroinflammation levels in the brain. It has been reported that [18F]LW223 is a suitable PET ligand for assessing TSPO abundance in the central nervous system (CNS) 60, 61. Alleviation of the neuroinflammatory response in the striatum of TDP-43 (M337V) monkeys was observed three months after transplantation of NILB-hiPSC, as demonstrated by PET scanning with [18F]LW223 (Figure 7A-C). Compared to the RT-Ctrl group, there were significantly increased GFAP (Figure 7D, E) and Iba1 (Figure S4A, B) positive cells in the striatum of TDP-43 (M337V) monkeys, indicating that overexpression of mutant TDP-43 in the striatum can induce excessive activation of glial cells. However, after treatment with NILB-hiPSCs, the number of both GFAP (Figure 7D, E) and Iba1 (Figure S4A, B) positive cells was significantly reduced whereas the number of NeuN-positive cells was increased (Figure S4C, D), indicating that NILB-hiPSCs treatment can alleviate excessive glial cell activation and reduce neuroinflammation levels induced by mutant TDP-43 in the striatum of monkeys.

## Discussion

In this study, we aimed to investigate the impact of transplanted NILB-hiPSCs on TDP-43-mediated neuropathology in monkey brains. Our findings demonstrate that NILB-hiPSCs can alleviate the limb movement difficulties in the TDP-43-injected monkeys, providing evidence of a possible restoration of neuronal function in the affected brain region. Additionally, we observed that neurons derived from NILB-hiPSCs have reached maturity and exhibit normal electric activity. Finally, we have found that the transplantation of NILB-hiPSCs could significantly reduce reactive gliosis and cytoplasmic accumulation of mutant TDP-43, indicating their ability to interact functionally with other cell types in the host brain region.

Previous studies have reported neuroprotective effects following transplantation of GDNF-secreting MSCs or human embryonic stem cell-derived astrocytes. However, clinical studies have only shown limited therapeutic outcomes 62, 63. While stem cells can be induced to differentiate into specific types of neurons *in vitro* and then transplanted *in vivo* to replace damaged neurons, this process can harm the synaptic connections during transplantation. Moreover, changes in the post-transplant microenvironment may also contribute to the death of the transplanted neurons 64. Cyclosporine has been reported to have direct effects on improving peripheral nerve regeneration or influencing this process 65, 66. In our prior experiments, we utilized differentiated stem cells alone with cyclosporine administration, but we did not observe any significant improvement on the motor function of the TDP-43-injected monkeys. Unlike other types of stem cells, the *in vivo* induction of differentiation of NILB-hiPSCs may provide an advantage potentially overcoming the limitations observed in earlier studies. This feature may help avoid harming synaptic connections during transplantation and ameliorate changes in the post-transplant microenvironment, thereby improving the survival of the transplanted neurons 39.

Mouse studies have shown that transplantation of NILB-hiPSCs can differentiate into motor neurons 39. The ability of NILB-hiPSCS to differentiate to mature neurons in the primate brains remains to be investigated. However, in our early experiments in monkeys, we did not observe differentiation of NILB-hiPSCs into motor neurons when injected into the TDP-43-damaged spinal cord (data not shown). We suspect that this may have been due to the excessive deterioration of the pathological microenvironment in the monkey spinal cord. Since mutant TDP-43 triggers neurodegeneration in various brain regions, we shifted our focus to the striatum, a relatively small brain region that can be easily defined. Damage to the monkey striatum can lead to limb movement difficulty and provide a platform for evaluating neurodegeneration-related phenotypes.

In the monkey striatum, transplanted NILB-hiPSCs mainly differentiated into mature neurons expressing NeuN and MAP2. Our findings support the notion that the fate of differentiation is largely dependent on the microenvironments present in the host brain region once the NILB-hiPSCs start to differentiate after transplantation. This is in line with previous studies demonstrating that the host brain region plays a crucial role in determining the type of cells that stem cells differentiate into* in vivo* 67.

However, we did not observe the expression of DARPP-32 in these neurons, suggesting that they do not fully differentiate into mature medium spiny neurons (MSNs). Several protocols have been used to generate MSNs from human stem cells 68-72. Most of these studies investigated the transplantation of human stem cells in the rodent models. Also, it has not been successful in using human stem cells to produce D1- and D2-MSNs in the brain 73. Several factors could explain this result. First, MSN maturation *in vivo* is an exceptionally slow process that may extend beyond our experimental window. Second, the adult brain environment lacks the high levels of developmental signals necessary to guide neurogenesis and maturation. Consequently, the transplanted cells likely receive insufficient sustained support to complete their differentiation. Finally, the transplantation procedure itself, combined with the challenging adult brain environment, may induce metabolic stress that further hinders maturation. Although further investigation is needed to determine the *in vivo* conditions or factors that can promote the differentiation of transplanted NILB-hiPSCs into MSNs in primate brains, an interesting finding in our study is that once NILB-hiPSCs mature into neurons, they are able to establish connections with host neuronal cells, which can alleviate TDP-43-mediated neuropathology. It is possible that during neuronal differentiation, more efficient cell-cell interactions are established, leading to improved function of the host neuronal cells.

It is important to note that the monkey models we studied expressed transgenic TDP-43 in the striatum and showed the cytoplasmic accumulation of TDP-43, a typical neuropathological feature that is seen in various brain regions under a variety of pathological conditions 74. Thus, alleviation of TDP-43-mediated pathology could have broad implications for the treatment of different types of neurodegenerative diseases, at least those displaying typical TDP-43 pathological hallmarks such as increased gliosis and cytoplasmic mislocation of TDP-43. Notably, we observed that cytoplasmic TDP-43 accumulation was reduced in surrounding cells in the presence of neurons derived from transplanted NILB-hiPSCs, which may be due to the restoration of normal cell-cell interactions by the transplanted neurons. Our findings also demonstrate that reactive gliosis was reduced by NILB-hiPSCs transplantation, supporting the idea that transplanted neurons restore normal cell-cell interactions and promote overall tissue health.

The promising results of our study are derived from a 3-month post-transplantation period, a duration strategically chosen to assess early neuronal maturation and integration. Although a longer observation window would more rigorously define the treatment's efficacy and safety profile, it introduces considerable challenges. These include the risks of delayed immune rejection and tumorigenicity, coupled with the significant animal resources and high costs inherent to non-human primate research. Consequently, while our findings are limited to this initial phase, they successfully establish the therapeutic potential of NILB-hiPSCs and provide a robust rationale for securing future funding and resources dedicated to long-term evaluation.

## Conclusion

In conclusion, transplantation of NILB-hiPSCs could effectively alleviate TDP-43-mediated neuropathology, including increased gliosis and cytoplasmic mislocalization of TDP-43, making it a potential treatment for different types of neurodegenerative diseases.

## Supplementary Material

Supplementary figures.

Supplementary movie.

## Abbreviations

TDP-43: TAR DNA-binding protein 43
NILB-hiPSCs: Multi-gene modified stem cells
ALS: Amyotrophic lateral sclerosis
NSCs: Neural stem cells
MSCs: Mesenchymal stem cells
GRPs: Glial-restricted progenitor cells
ESCs: Embryonic stem cells
hiPSCs: Human induced pluripotent stem cells
GDNF: Glial cell line-derived neurotrophic factor
PD: Parkinson's disease
Tet-On: tetracycline-inducible expression system
