# Supplementary material for: Alleviation of mutant TDP-43-mediated neuropathology by inducible stem cells in monkeys
Source: Int J Biol Sci. 2026 Jan 1;22(1):25–42. doi: 10.7150/ijbs.122557 (PMC12681693; doi:10.7150/ijbs.122557)
Supplement: Supplementary file 1 — Supplementary figures. [file ijbsv22p0025s1.pdf]

Supplementary Materials

Alleviation of mutant TDP-43-mediated neuropathology by inducible stem cells in monkeys

Authors: Xichen Song[1,#], Caijuan Li[1,#], Yang Yang[2,#], Chunhui Huang[1,#], Min Chen[3], Song Lin[4], Zhonghai Huang[1], Wei Wang[1], Kai Liao[5], Huiyi Wei[5], Lu Wang[5], Hao Xu[5], Yizhi Chen[1], Yingqi Lin[1], Jiawei Li[1], Zhen Dai[2], Wenguang Xie[2], Xiao Zheng[1], Jianhao Wu[1], Jiale Gao[1], Jiaxi Wu[1], Zhuchi Tu[1], Libing Zhou[1], Lu Huang[1], Chaoran Ren[1], Kwok-Fai So[1], Peng Yin[1], Huiming Yang[6], Shihua Li[1], Liangxue Lai[2], Xiao-Jiang Li[1,7], Sen Yan[1,✉]

Affiliations:

[1] State Key Laboratory of Bioactive Molecules and Druggability Assessment, Guangdong Basic Research Center of Excellence for Natural Bioactive Molecules and Discovery of Innovative Drugs, Guangdong Provincial Key Laboratory of Non-human Primate Research, Guangdong-Hong Kong-Macau Institute of CNS Regeneration, School of Medicine, Jinan University, Guangzhou,510632, China.

[2] Guangdong Provincial Key Laboratory of Stem Cell and Regenerative Medicine, Guangzhou Institutes of Biomedicine and Health, Chinese Academy of Sciences, Guangzhou, 510632, China.

[3] South China Institute of Large Animal Models for Biomedicine, Wuyi University, Jiangmen, 529000, China.

[4] Department of Physiology School of Medicine, Jinan University, Guangzhou, 510632, China.

[5] The First Affiliated Hospital of Jinan University, Guangzhou, 510632, China.

[6] Sun Yat-sen University, Guangdong Provincial Key Laboratory of Diagnosis and Treatment of Major Neurological Diseases, Guangzhou, China.

[7] Lingang Laboratory, Shanghai, 201306, China.

# These authors contributed equally to this work.

Corresponding author at: Sen Yan, GHM Institute of CNS Regeneration, School of Medicine, Jinan University, No.601 West Huangpu Avenue, Tianhe District, Guangzhou Guangdong Province， 510623 China. E-mail: 231yansen@163.com.

Supplementary Figures

Supplementary Figure 1

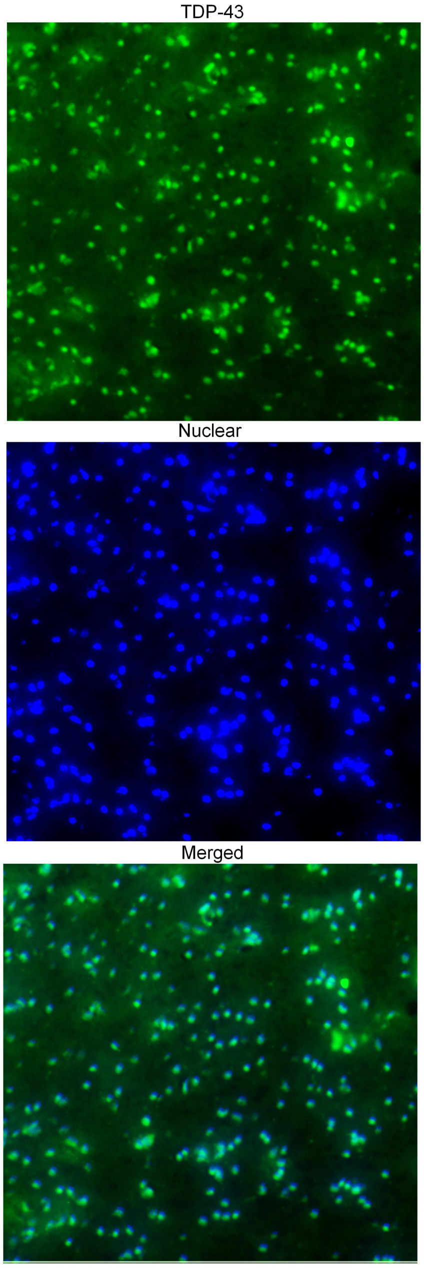

Supplementary Figure 1. Nuclear distribution of endogenous TDP-43 in the monkey brain

The striatum of wild type monkey was stained with the anti-TDP-43 (G400). The nucleus were labeled by DAPI.

Supplementary Figure 2

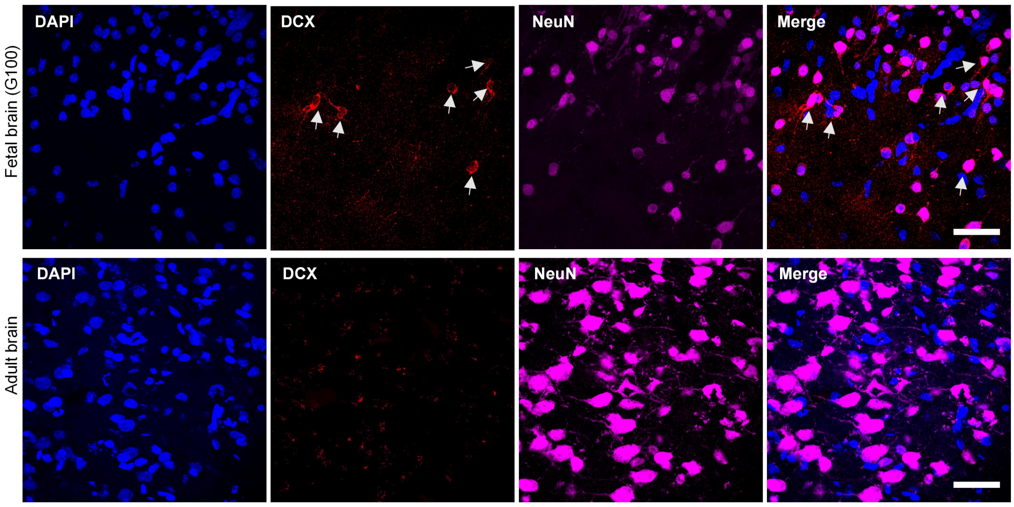

Supplementary Figure 2. DCX expression (arrows) is minimal in the cortical regions of fetal monkey brains at approximately 100 days of gestation (~G100) and undetectable in adult monkeys aged seven year

The cortical regions of ~G100 and adult monkey were stained with the anti-DCX and anti-NeuN antibodies, The nuclei were labeled by DAPI. Scale bar, 40 μm.

Supplementary Figure 3

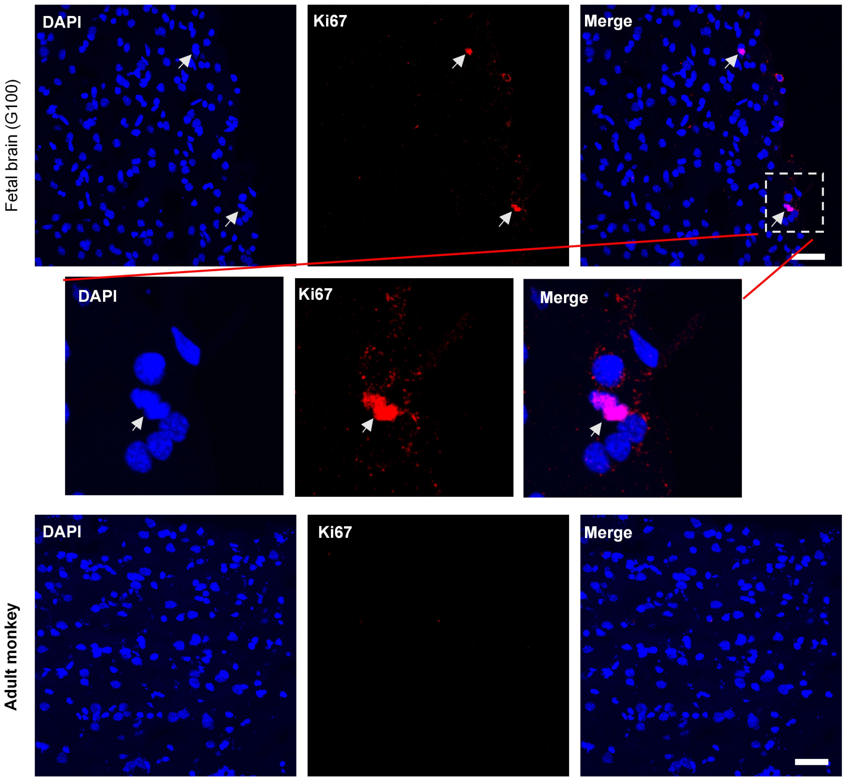

Supplementary Figure 3. Ki67 expression (arrows) is minimal or undetectable in the cortical regions of fetal monkey brains at approximately 100 days of gestation (~G100) and in adult monkeys aged seven years

The cortical regions of ~G100 and adult monkey were stained with the anti-Ki67 antibodies, The nuclei were labeled by DAPI. Scale bar, 40 µm.

Supplementary Figure 4

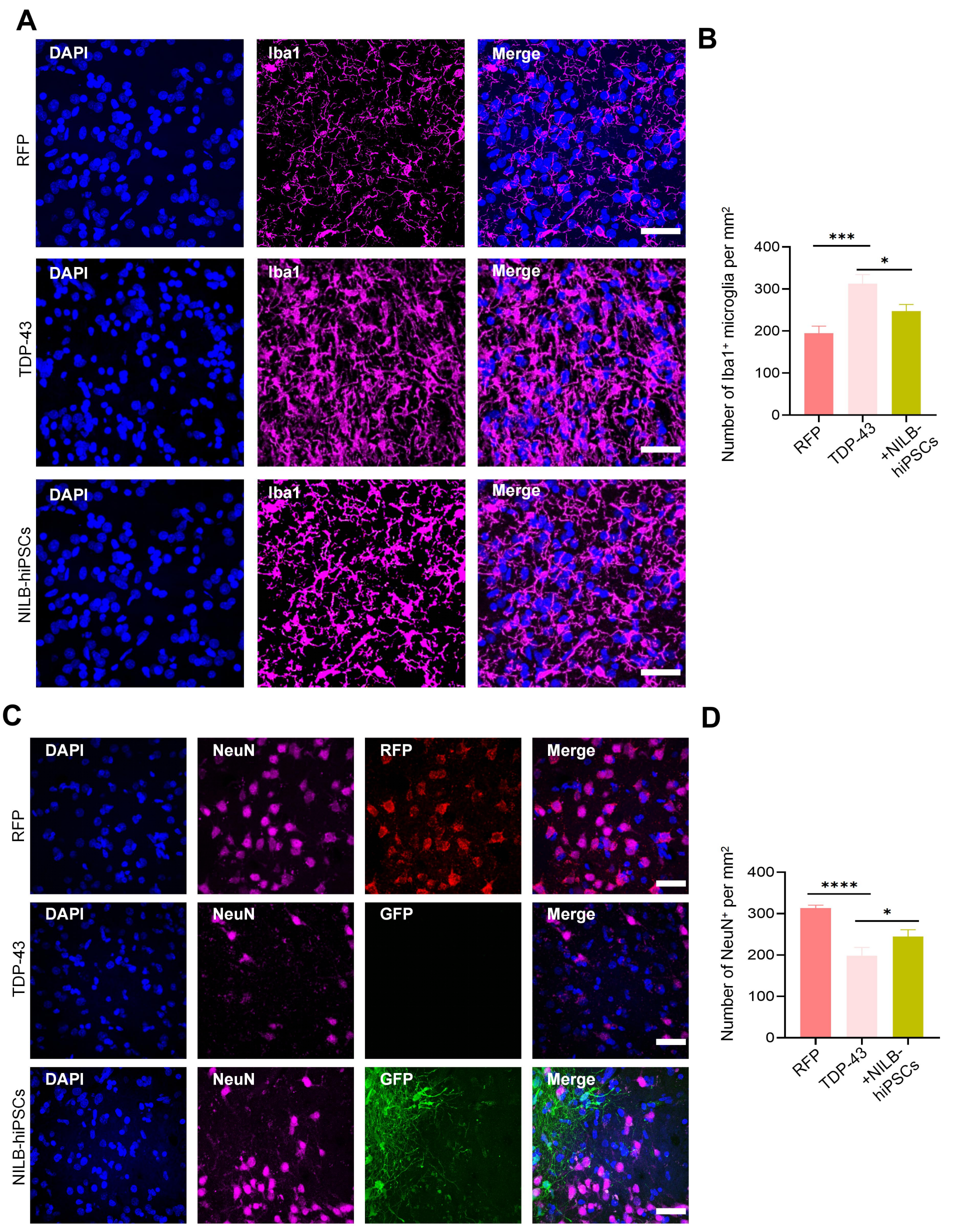

Supplementary Figure 4. NILB-hiPSCs treatment effectively reduces neuroinflammation levels and mitigates neuronal loss

(A) Representative immunofluorescent fluorescent images of the striatum form TDP-43 or RFP injected monkey. Antibodies for Iba1 and DAPI were used. Scale bar, 40 µm. (B) Quantitative assessment of Iba1-positive cells. (C) Representative immunofluorescent fluorescent images of the striatum from AAV-TDP-43 or RFP injected monkey. Antibodies for NeuN, GFP, RFP, DAPI were used. (D) Quantitative

assessment of NeuN-positive cells . One-way ANOVA revealed statistical significance (*$P < 0.05$, **$P < 0.01$, ****$P < 0.0001$). Data are presented as mean ± SEM (n=3). Scale bars, 40 μm.
